# Supplementary material for: The Anemia Stress Index—Anemia, Transfusions, and Mortality in Patients with Continuous Flow Ventricular Assist Devices
Source: J Clin Med. 2022 Aug 3;11(15):4517. doi: 10.3390/jcm11154517 (PMC9369576; doi:10.3390/jcm11154517)
Supplement: Supplementary file 1 [file jcm-11-04517-s001.zip › jcm-1731652-supplementary.pdf]

Supplemental Table S1: Results of Cox Regression model examining association between ASI at 24 hours post LVAD implant and time to mortality

Variable	HR (95% CI)
ASI at 24 hours	1.08 (1.03 – 1.14)
Age at Implant, per year increase	1.03 (1.00-1.05)
Male Sex	0.78 (0.37-1.63)
Pre-Implant Body Mass Index, per unit increase	1.07 (1.02-1.12)
Diabetes Mellitus	0.62 (0.35-1.09)
Chronic Obstructive Pulmonary Disease	1.04 (0.57-1.97)
History of smoking	1.50 (0.85-2.67)
Atrial fibrillation	0.77 (0.44-1.34)
Pre-Implant Chronic Kidney Disease	2.21 (1.27-3.85)
Previous sternotomy	0.63 (0.36-1.10)
Indication for LVAD Implantation<br>Bridge to Transplant<br>Destination Therapy<br>Bridge to Decision<br>Bridge to Recovery	 Reference<br>2.44 (0.93-6.42)<br>0.70 (0.64-2.53)<br>-*
Type of LVAD implanted<br>HM2<br>HM3	 Reference<br>3.03 (1.26-7.29)

* estimate not calculated due to very small number of patients.
